# Spatial spread of *Wolbachia* in *Rhagoletis cerasi* populations

**DOI:** 10.1098/rsbl.2018.0161

**Published:** 2018-05-23

**Authors:** Vid Bakovic, Martin Schebeck, Arndt Telschow, Christian Stauffer, Hannes Schuler

**Affiliations:** 1 Department of Forest and Soil Sciences, Boku, University of Natural Resources and Life Sciences, Vienna, Austria; 2 Institute for Evolution and Biodiversity, University of Münster, Münster, Germany; 3 Laimburg Research Centre, Pfatten, Italy

**Keywords:** endosymbiont, European cherry fruit fly, modelling, cytoplasmic incompatibility

## Abstract

The bacterial endosymbiont *Wolbachia* has been used to control insect pests owing to its ability to manipulate their life history and suppress infectious diseases. Therefore, knowledge on *Wolbachia* dynamics in natural populations is fundamental. The European cherry fruit fly, *Rhagoletis cerasi*, is infected with the *Wolbachia* strain *w*Cer2, mainly present in southern and central European populations, and is currently spreading into *w*Cer2-uninfected populations driven by high unidirectional cytoplasmic incompatibility. Here, we describe the distribution of *w*Cer2 along two transition zones where the infection is spreading into *w*Cer2-uninfected *R. cerasi* populations. Fine-scale sampling of 19 populations in the Czech Republic showed a smooth decrease of *w*Cer2 frequency from south to north within a distance of less than 20 km. Sampling of 12 Hungarian populations, however, showed a sharp decline of *w*Cer2 infection frequency within a few kilometres. We fitted a standard wave equation to our empirical data and estimated a *Wolbachia* wave speed of 1.9 km yr^−1^ in the Czech Republic and 1.0 km yr^−1^ in Hungary. Considering the univoltine life cycle and limited dispersal ability of *R. cerasi*, our study highlights a rapid *Wolbachia* spread in natural host populations.

## 1. Introduction

*Wolbachia* is an endosymbiotic bacterium that is present in a wide range of arthropod and nematode species and can alter the reproduction of its host [1]. Being maternally inherited, *Wolbachia* is able to modify the reproduction of its host to its own advantage. The most efficient way is the induction of cytoplasmic incompatibility (CI) which results in embryonic mortality when the sperm of an infected male fertilizes the egg of a female that is not infected or is infected with a different *Wolbachia* strain [2]. Infected females, in contrast, produce viable offspring with both infected and uninfected males. This results in a reproductive advantage of infected over uninfected females and facilitates the spread of *Wolbachia* through host populations [3]. Although horizontal transmission within and among species is possible [4–6], *Wolbachia* mainly spreads vertically from females to their offspring via the egg cytoplasm [7].

Predicting *Wolbachia* spread through natural host populations is of considerable importance to understand how this bacterium invades new territory. Important parameters that influence the infection dynamics of *Wolbachia* are the strength of CI, the efficacy of maternal transmission, fitness effects on its host and the reproductive and dispersal potential of its host species [8]. The spread of the *Wolbachia* strain *w*Ri in *Drosophila simulans* in California [3] and *w*Au in the same species over the eastern coast of Australia [8] are the best-studied examples of rapid *Wolbachia* spread in natural populations. These studies show that *Wolbachia* is able to provide fitness benefits to its host, enhancing the spatial spread from low initial infection frequencies [8]. By contrast, fecundity costs can prevent a range expansion of the endosymbiont. In this case, *Wolbachia* spreads as a bistable wave where a certain threshold frequency is necessary to get established [9]. This has been shown in *Aedes aegypti* artificially transinfected with *w*Mel, where the *Wolbachia* infection causes fitness costs to its host that limit the spread of released populations [10].

The European cherry fruit fly, *Rhagoletis cerasi*, is an important agricultural pest of cherries that is distributed throughout Europe [11]. This tephritid is infected with at least five different *Wolbachia* strains [12,13]. All populations share one common strain, *w*Cer1, whereas a second strain, *w*Cer2, is mainly present in southern and central European populations [12]. This strain causes a high degree of CI between *w*Cer2-infected males and *w*Cer2-uninfected females, with egg mortality rates of up to 98% [14], and is currently spreading in central Europe [15].

The cherry fruit fly system provides an excellent model to study the invasion dynamics of *Wolbachia*: First, *R. cerasi* has a univoltine life cycle that allows an in-depth characterization of an ongoing spatial *Wolbachia* spread in natural populations. Second, dispersal rates of the fly are limited, with an average estimate of 200 m yr^−1^ and a few long distance dispersers migrating about 4 km yr^−1^ [16]. This allows the study of *Wolbachia* range expansion on a small geographical scale. Here, we characterize the infection frequency of *w*Cer2 along two transition zones: one along a south–north axis in the Moravian region of the Czech Republic and the other along a west–east axis found in northern Hungary. We use a standard Barton–Turelli wave model [9] to estimate *R. cerasi* adult dispersal potential and approximate the width and speed of the *Wolbachia* travelling wave. Our results highlight a rapid ongoing *Wolbachia* spread in natural populations of *R. cerasi*.

## 2. Material and methods

### (a) Collection and genetic analysis

**Figure 1.**
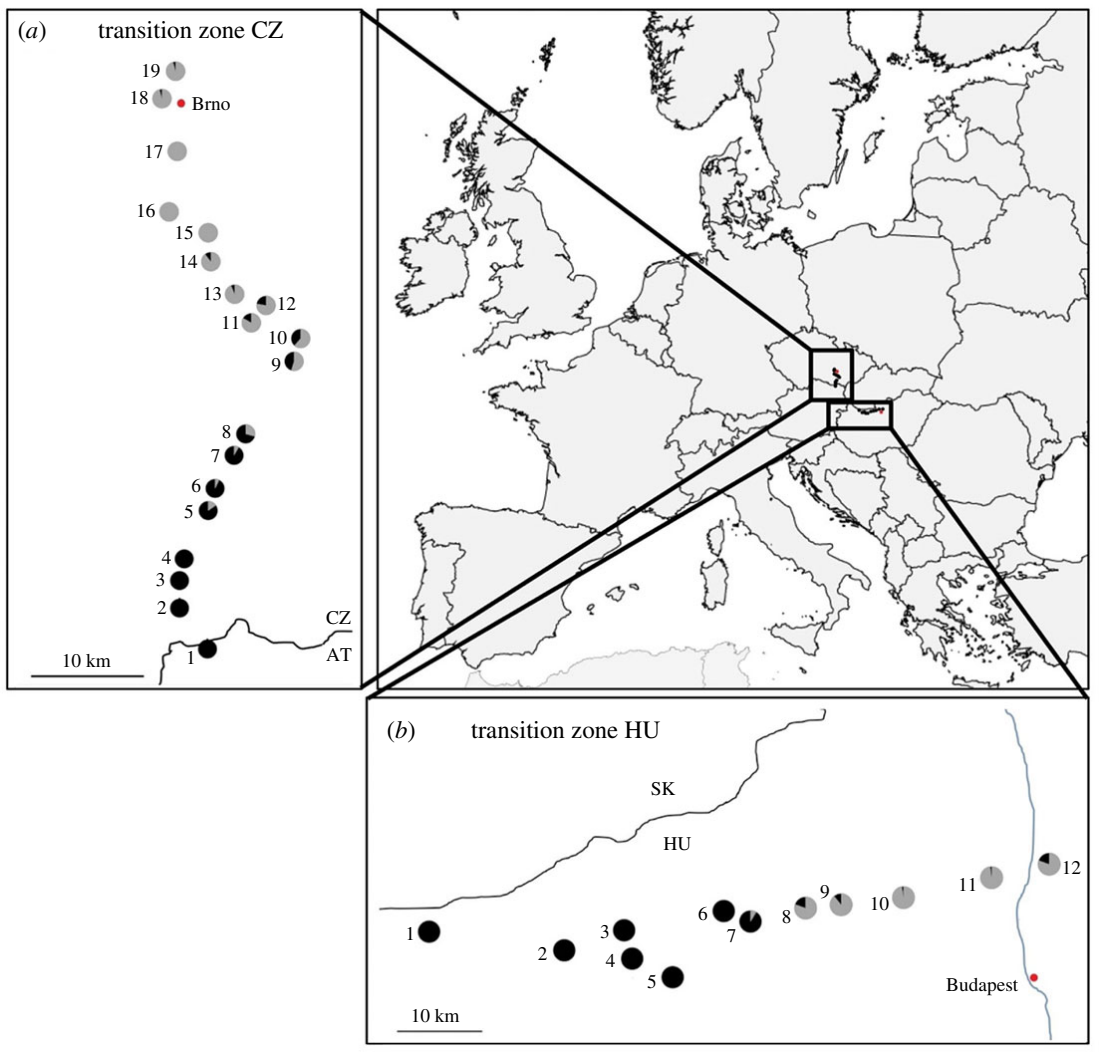
Geographical location and spatial distribution of the two *w*Cer2 transition zones of *Rhagoletis cerasi*. Each pie chart represents a single *Prunus avium* tree with ratio of *w*Cer2-infected (black) and *w*Cer2-uninfected (grey) individuals of *R. cerasi* in the Czech transition zone in 2015 (*a*) and in the Hungarian transition zone in 2016 (*b*). (Online version in colour.)

Larvae and pupae of *R. cerasi* were collected in 2015 in Austria and the Czech Republic and in 2016 in Hungary from *Prunus avium.* All populations from each transect were sampled on the same day and each population was sampled from a single tree. Samples were stored in absolute ethanol at −20°C. Five hundred and forty-eight individuals of *R. cerasi* were collected along a south–north transect of 46 km from one population in Austria (CZ-1) and 18 populations in the Czech Republic (CZ-2 to CZ-19). Furthermore, 336 individuals were collected from 12 populations in Hungary (HU-1 to HU-12) along a 72 km west–east transect (figure 1; electronic supplementary material, S1). DNA was extracted using the GenElute Mammalian Genomic DNA miniprep kit (Sigma-Aldrich, St Louis, MO, USA). *Wolbachia* screening was performed on all collected samples using diagnostic polymerase chain reaction (PCR) with strain-specific primers targeting specific fragments of the *Wolbachia* surface protein gene (*wsp*) [13]. Since *w*Cer1 is fixed in *R. cerasi, w*Cer1-specific primers were used as positive controls for DNA quality.

### (b) Wave parameters

The spatial distribution of *Wolbachia* was modelled by a standard nonlinear equation that describes *Wolbachia* spread as a traveling wave [3]. Using the standard wave model, we estimated the adult fly dispersal potential and the *Wolbachia* wave width and speed in the Czech Republic and in Hungary (see details in electronic supplementary material, S2).

## 3. Results

### (a) *w*Cer2 infection frequencies

In the Czech transition zone, the four southernmost populations CZ-1 to CZ-4 were completely *w*Cer2-infected. Populations CZ-5 and CZ-6 had high *w*Cer2 infection frequencies of 85 and 93%, respectively. Similarly, 92% of the individuals from CZ-7 and 71% of the individuals from CZ-8 were *w*Cer2-infected. A medium infection frequency was found in populations CZ-9 and CZ-10, with a *w*Cer2 infection rate of 45 and 38%*,* respectively. Relatively low infection frequencies were found in CZ-11, with 17%, CZ-12 with 22%, CZ-13 with 6% and CZ-14 with 10% *w*Cer2-infected individuals, and three populations further north (CZ-15 to CZ-17) were completely *w*Cer2-uninfected. The two northernmost populations, CZ-18 and CZ-19, however, showed a *w*Cer2 infection frequency of 4%, i.e. two *w*Cer2-infected individuals (*n* = 48) in each population (figure 1; electronic supplementary material, S1).

In the Hungarian transition zone, the six westernmost populations (HU-1 to HU-6) were completely *w*Cer2-infected. In HU-7 91% of the individuals were *w*Cer2-infected, while 7 km further east in HU-8, *w*Cer2 was present in just 20% of the individuals. In HU-9 a *w*Cer2 infection rate of 11% was found, while HU-10 and HU-11 both had a low infection frequency of 2%. In the most eastern population, HU-12, 20% of the individuals were *w*Cer2-infected (figure 1 and electronic supplementary material, S1).

### (b) Wave parameters

**Figure 2.**
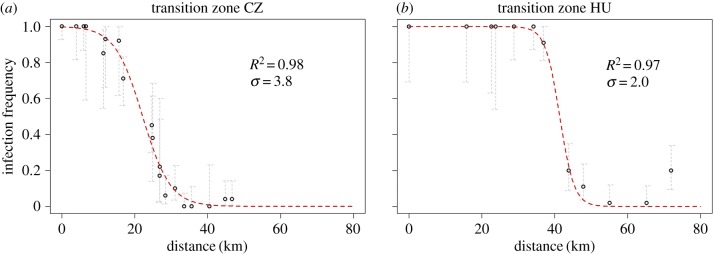
Predicted and observed *w*Cer2 infection frequencies in the two transition zones of *R. cerasi* in the Czech Republic (*a*) and Hungary (*b*). Grey bars represent the 95% confidence intervals of infection frequency. (Online version in colour.)

A nonlinear least-squares fit showed that the standard wave model fits well to our *w*Cer2 infection frequency data with an *R*^2^ value of 0.98 in the Czech Republic and 0.97 in Hungary (figure 2). *Rhagoletis cerasi* adult dispersal (*σ*) estimated from the least-squares best fit was 3.8 km in the Czech Republic and 2.0 km in Hungary (figure 2). By using these two dispersal parameter values we estimated a wave width of 11.4 km and a wave speed of 1.9 km yr^−1^ in the Czech Republic, while in Hungary, we estimated a wave width of 5.8 km and a wave speed of 1.0 km yr^−1^.

## 4. Discussion

The classical textbook examples of *Wolbachia* spread in nature are the invasion of *w*Ri in *D. simulans* populations in California [3] and Australia [8], and the release of artificially *Wolbachia*-infected *Ae. aegypti* mosquitos in Australia [10,17]. These studies showed how different *Wolbachia-*induced fitness effects influence its establishment and spread: while fitness costs hinder low-frequency infections from establishment and spread of *Wolbachia* in *Ae. aegypti* populations [10], positive fitness effects of *Wolbachia* resulted in a rapid spread of *w*Ri in *D. simulans* of about 100 km yr^−1^ [3,8]. The dispersal rate of *w*Mel in artificially transinfected mosquitoes in Australia, however, was multiple orders of magnitude lower, with a spatial spread of 100–200 m yr^−1^ [10].

The *Wolbachia* strain *w*Cer2 has been shown to spread in *R. cerasi* within Central Europe, where the infection dynamics of its invasion were determined on a large scale [15]. Here we characterized the *w*Cer2 frequency and estimated its spatial spread along two fine-scale transects of *R. cerasi*. We estimated an *R. cerasi* adult dispersal rate of 3.8 km per generation in the Czech Republic and 2.0 km per generation in Hungary. Differences in the estimated migration rates might have been influenced by dissimilarities in the landscape and the presence of hosts between the two transects. The estimated adult dispersal rate is in line with a capture–release maximal dispersal estimation of 4 km per generation [16]. We estimated a wave width of 11.4 km in the Czech Republic and 5.8 km in Hungary. This is in stark contrast to an estimated wave width of 170–260 km in a German *w*Cer2 transition zone that might be influenced by long-dispersal migration of the fly [15]. The *Wolbachia* wave speed was estimated to be 1.9 km yr^−1^ in the Czech Republic and 1.0 km yr^−1^ in Hungary. Considering the univoltine biology of the fly, *w*Cer2 is spreading with a rate of 1.9 km per generation in the Czech Republic and 1.0 km per generation in Hungary.

The infection frequency of *w*Cer2 in the Hungarian transition zone has already been studied, in 1999, [12] and allowed a direct comparison with our data from 2016. Since the wave speed is defined as the distance travelled by an intermediate infection frequency over time (e.g. 50% infection rate), we measured the longitudinal distance between populations infected with greater than 50% in 1999 (HU-1; [12]) and 2016 (HU-7). HU-1 was 100% infected in 1999 while in 2016 *w*Cer2 was present in 91% of the individuals from HU-7, 36 km further east, resulting in an estimated wave speed of 2.0 km yr^−1^. Although, we consider that this rough estimation might have overestimated the spread of *w*Cer2 in Hungary, this direct comparison supports our estimated fast spatial spread of *Wolbachia.* Repeated fine-scale samplings over different years are needed to refine the estimated temporal and spatial dynamics of the *w*Cer2 spread. In summary, considering the univoltine biology and the low dispersal rate of this fly, our study represents a new example of a rapid *Wolbachia* spread in nature.

## Supplementary Material

ESM1

## Supplementary Material

ESM2

## Supplementary Material

ESM3

## Data accessibility

Data are provided in the electronic supplementary material.
