# Supplementary material for: Spatial spread of Wolbachia in Rhagoletis cerasi populations
Source: Biol Lett. 2018 May 23;14(5):20180161. doi: 10.1098/rsbl.2018.0161 (PMC6012700; doi:10.1098/rsbl.2018.0161)
Supplement: ESM2 [file rsbl20180161supp2.docx]

**Electronic Supplementary Material (ESM), S2: Materials and methods for Bakovic V, Schebeck M, Telschow A, Stauffer C, Schuler H “Spatial spread of *Wolbachia* in *Rhagoletis cerasi* populations” Biology Letters**

*Wave parameters*

We followed [1,2,3] and estimated the infection dynamics of *w*Cer2 by a nonlinear equation. The spread of *Wolbachia* has been described as a travelling wave approximated by:

$p\left( x,t \right)=\frac{1}{2}\left[ 1-tanh\left( \frac{x-\sigma\sqrt{lCI}t/2}{2\sigma/\sqrt{lCI}} \right) \right]$ (1)

where p(x,t) is the infection frequency at point x at time t, (σ√l_CI_)/2 is the wave speed, σ is a measure of the average adult migration distance, l_CI_ is the strength of CI (0.98 according to [4]), and w=σ/√l_CI_ is the wave width, such that the infection frequency rises from 5% to 95% over ∆x = 3w. The function holds true under the assumption that *Wolbachia* does not have fitness effects on its host and that it has 100% maternal transmission rate [5].

We tested how well this model fits to our empirical data at one time-point. A best fitting σ (average adult migration) value was derived by conducting a least squares best fit of the equation to our data, while allowing σ to vary. Because we fit this model to our data in order to analyse the shape of the *w*Cer2 wave front, we arbitrarily used the best fitting t (t moves the wave left and right along the x axis as a function of time). Incorporating σ, we calculated the wave width as (w=σ/√l_CI_ | ∆x = 3w) and the wave speed as (σ√l_CI_)/2. Curve fitting was conducted using the *nls* function in R [6] and is provided in electronic supplementary material S3.

**References**

1. Barton NH. 1979 The dynamics of hybrid zones. *Heredity,* **43**, 341-359. (doi: 10.1038/hdy.1979.87)

2. Turelli M, Hoffmann AA. 1991 Rapid spread of an inherited incompatibility factor in California Drosophila. *Nature* **353**, 440–442. (doi:10.1038/353440a0)

3. Schofield P. 2002 Spatially explicit models of Turelli-Hoffmann *Wolbachia* invasive wave fronts. *J. Theor. Biol.*, **215**, 121-131. (doi:10.1006/jtbi.2001.2493)

4. Boller E, Bush GL. 1974 Evidence for genetic variation in populations of the European cherry fruit fly, *Rhagoletis cerasi* (Diptera: Tephritidae) based on physiological parameters and hybridization experiments. *Entomol. Exp. Appl.* **17**, 279–293. (doi:10.1111/j.1570-7458.1974.tb00345.x)

5. Schuler H. et al. 2016 The hitchhiker's guide to Europe: the infection dynamics of an ongoing *Wolbachia* invasion and mitochondrial selective sweep in *Rhagoletis cerasi*. *Mol. Ecol.* **25**, 1595–1609. (doi:10.1111/mec.13571)

6. R Core Team. 2017 R: A Language and Environment for Statistical Computing. Vienna, Austria: R Foundation for Statistical Computing. (See http://www.R-project.org)
